# 2-step reaction kinetics for hydrogen absorption into bulk material via dissociative adsorption on the surface

**DOI:** 10.1038/s41598-021-98347-4

**Published:** 2021-09-22

**Authors:** Taro Yakabe, Gaku Imamura, Genki Yoshikawa, Naoya Miyauchi, Masahiro Kitajima, Akiko N. Itakura

**Affiliations:** 1 Research Center for Advanced Measurement and Characterization, National Institute for Materials Science (NIMS), 1-2-1 Sengen, Tsukuba, Ibaraki 305-0047 Japan; 2 International Center for Materials Nanoarchitectonics, National Institute for Materials Science (NIMS), 1-1 Namiki, Tsukuba, Ibaraki 305-0044 Japan; 3 Center for Functional Sensor & Actuator, National Institute for Materials Science (NIMS), 1-1 Namiki, Tsukuba, Ibaraki 305-0044 Japan

**Keywords:** Surfaces, interfaces and thin films, Reaction kinetics and dynamics, Hydrogen storage materials, Sensors

## Abstract

We have demonstrated that the process of hydrogen absorption into a solid experimentally follows a Langmuir-type (hyperbolic) function instead of Sieverts law. This can be explained by independent two theories. One is the well-known solubility theory which is the basis of Sieverts law. It explains that the amount of hydrogen absorption can be expressed as a Langmuir-type (hyperbolic) function of the square root of the hydrogen pressure. We have succeeded in drawing the same conclusion from the other theory. It is a 2-step reaction kinetics (2sRK) model that expresses absorption into the bulk via adsorption on the surface. The 2sRK model has an advantage to the solubility theory: Since it can describe the dynamic process, it can be used to discuss both the amount of hydrogen absorption and the absorption rate. Some phenomena with absorption via adsorption can be understood in a unified manner by the 2sRK model.

## Introduction

Hydrogen is the smallest element and a hydrogen ion H^+^ can be thought of as a point charge. If the size of neutral H^0^ is regarded as the Bohr radius, the size is about 0.05 nm. It is also known that hydrogen molecules are adsorbed dissociatively onto the surfaces of some kinds of materials and the hydrogen atoms then are absorbed into the bulk of them. The positive aspect of this phenomenon is that these materials can be used as storage materials^1^, hydrogen filters^2^, or sensors^3,4^; the negative aspect is that it may cause embrittlement of some materials and lead to their destruction. Sieverts’ law is the most well-known theory of hydrogen absorption into solids. It is obtained from solubility theory in thermodynamics, and is expressed as

1 $$\frac{\theta }{1-\theta }={\left(\frac{{P}_{H2}}{P(T)}\right)}^{1/2},$$

where *θ* is the ratio of the number of absorbed hydrogen atoms per the number of sites capable of absorption*, P*_*H2*_ is the hydrogen gas pressure, and *P*(*T*) is a constant with a pressure dimension that depends on temperature *T*^5,6^. When the hydrogen concentration in the solid is low enough (*θ* <  < 1), the left side of Eq. (1) becomes *θ*, which is Sieverts’ law. Solving it for *θ* without the approximation yields a Langmuir-type (hyperbolic) function. The Langmuir-type function appears in an ideal molecular adsorption on surface.

In our previous work, we demonstrated the detection of hydrogen by using a simple Pd film with a membrane-type surface stress sensor (MSS)^7^. The MSS is an optimized nanomechanical sensor which can measure mechanical deformation to nanometre precision and be used for observing various target analytes by selecting a sensitive film^8^. We confirmed that the sensing signals of hydrogen absorption decreased with the number of absorption and desorption cycles because of the hysteresis behaviour of the cycles of them in the Pd–H system^9^. By focusing on the hydrogen absorption rate (the time derivative of the absorption amount), we clarified experimentally that the absorption rate was fitted by a Langmuir-type function of the square root of the hydrogen concentration. Furthermore, the relation was proved theoretically as well with a 2-step reaction kinetics (2sRK) model.

Considering the application as a sensor, short reaction and recovery times and reproducibility are important for hydrogen sensor. In particular, it is desirable that the reaction time is one second to a few ten seconds^3,10^. In the current study, we employed an amorphous Pd alloy, which has been studied as the sensing material in other types of hydrogen sensors owing to its low hysteresis and fast response^11,12^. With this alloy, we succeeded in demonstrating hydrogen sensing with low hysteresis and fast response. Our experimental results showed that the amount of the absorbed hydrogen was fitted by a Langmuir-type (hyperbolic) function of the square root of the hydrogen concentration instead of Sieverts’ law. We show in this paper that this relationship can be also explained by the 2sRK model we developed.

## Experimental results and discussion

**Figure 1:**
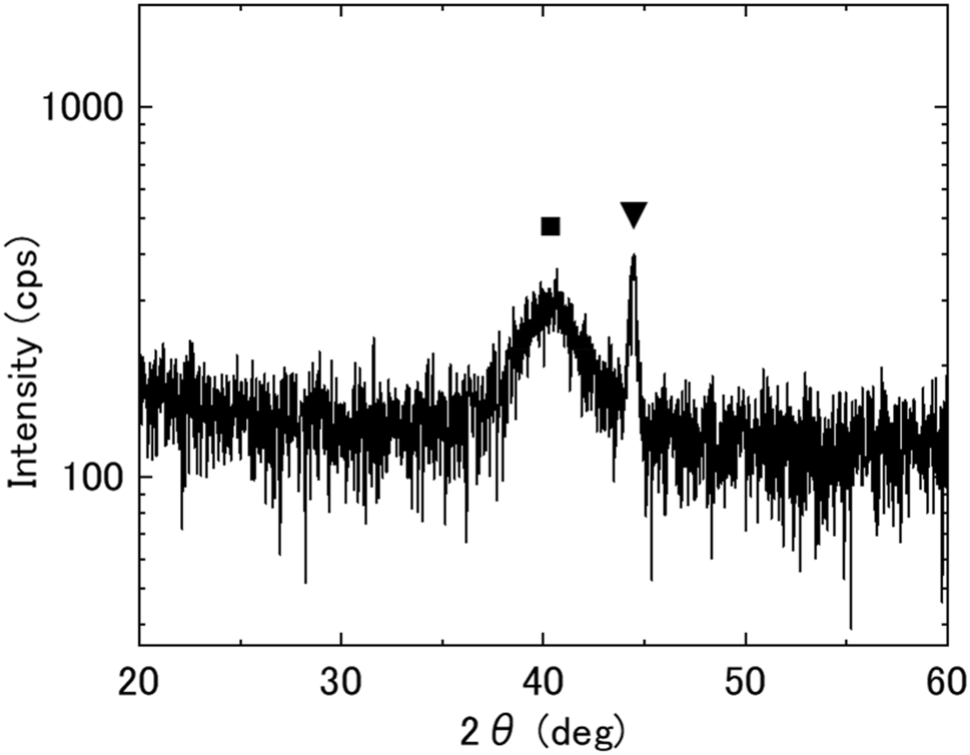
XRD profile of Pd_75_Cu_10_Si_15_ thin film on Si(100). There are two peaks: a broad peak (■) around 40 degrees from the amorphous structure and a sharp peak (▼) around 45 degrees from partial crystallization of Pd(200) or γ-PdCu.

The principle of hydrogen detection using a hydrogen storage material and a stress sensor is that when the material stores hydrogen, the interatomic distance spreads and stress is generated, which is transmitted to the stress sensor and output. In this study, we used Pd_75_Cu_10_Si_15_ as a sensitive film for hydrogen sensing. The Pd_75_Cu_10_Si_15_ film was made by co-sputtering of the elements at room temperature onto the MSS. The thicknesses of the sensitive films on the MSS were set to 30 nm and 50 nm. For structural analysis of the film, we made a 50 nm film on a Si(100) substrate. The structure of the Pd_75_Cu_10_Si_15_ film was analysed by X-ray diffraction (XRD; Rigaku Smart Lab) at room temperature (Fig. 1). There were two peaks: a broad peak around 40 degrees from the amorphous structure and a sharp peak around 45 degrees from the partial crystallization of Pd(200)^13^ or γ-PdCu^14^. In our hydrogen measurement system^7^, the hydrogen concentration is adjusted by mixing the hydrogen source gas with pure nitrogen gas (99.999%). We used two kinds of hydrogen source gas: 40 000 ppm (4.00%) ± 100 ppm (0.01%) and 100 ppm ± 10 ppm. Two mass-flow controllers (SEC-N112MGM; Horiba STEC) were used to adjust the hydrogen concentration flowing into the measurement chamber in which the MSS coated with the Pd_75_Cu_10_Si_15_ film was placed. Since the flowing gas was discharged without pumping, the total pressure of the mixture gas in the vicinity of the MSS was considered to be atmospheric pressure. The temperatures of the MSS measurement chamber and gas line were maintained at 298.0 ± 0.1 K. The stress signals were read as electrical voltages of the MSS with sampling times of 1 s (i.e.*,* 1-Hz measurement).

**Figure 2:**
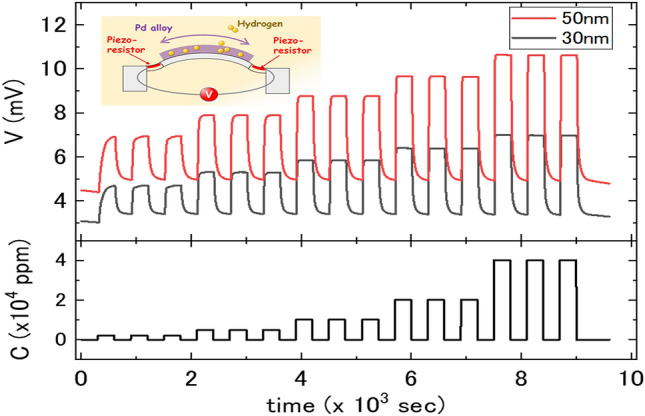
Responses of PdCuSi/MSS to hydrogen concentrations of 2000, 4800, 10 000, 20 000, and 40 000 ppm. Their baselines are not corrected (no offset). Horizontal axis represents time, and vertical axes represent the signals of the MSS (V) and the concentration of hydrogen (C) regulated by the mass-flow controller. Inset: Schematic diagram of stress output as piezo voltage by the MSS when hydrogen atoms are absorbed into the amorphous Pd-alloy film.

Figure 2 shows the raw MSS signals for hydrogen concentrations of 2000, 4800, 10,000, 20,000, and 40,000 ppm. When hydrogen atoms are absorbed into the sensing film on the MSS, the induced mechanical stress of the film is transduced into an electrical signal via piezoresistors embedded in the narrow bridges that suspend the centre membrane^8^. Baselines reflect the internal stress of the Pd films and depend on the film thickness. Three cycles of 5 min hydrogen injection and 5 min nitrogen purge were executed for each concentration. The reaction time to saturation of 40,000 ppm was approximately 10 s, and the peak heights of the signals had good reproducibility. In the case of an MSS coated with a simple Pd film, it took 1 h or more to reach saturation and reproducibility was low^7^. It takes a little longer to saturate in the first absorption process for 2000 ppm in Fig. 2. The structure of the sensitive film should change slightly due to the first absorption and release process. Each release process is slower than each absorption one because the absorption process is an exothermic reaction and the release one is an endothermic reaction^11^. These results demonstrate the much faster hydrogen reaction in Pd_75_Cu_10_Si_15_ than in simple Pd and the good reproducibility for hydrogen absorption and release. We consider that the hydrogen diffusion was faster because hydrogen atoms can move easily along extended paths in the amorphous structure of the film.

**Figure 3:**
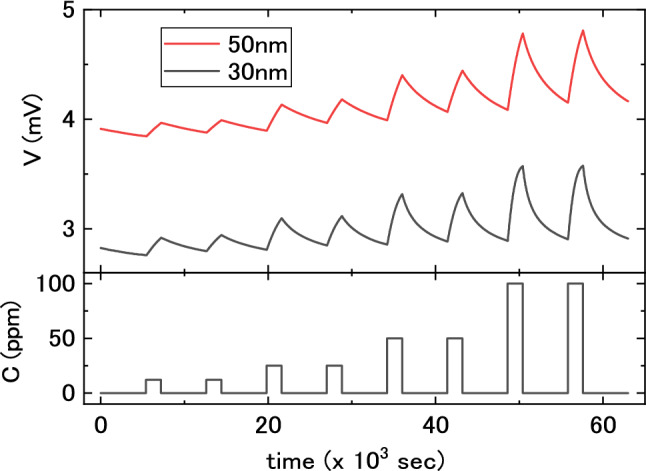
Responses of PdCuSi/MSS to hydrogen concentrations of 12, 25, 50, and 100 ppm. Their baselines are not corrected (no offset). Horizontal axis represents time, and vertical axes represent the signals of the MSS (V) and the concentration of hydrogen (C) regulated by the mass-flow controllers.

Figure 3 shows the raw MSS signals for lower hydrogen concentrations of 12, 25, 50, and 100 ppm. Two cycles of 30 min hydrogen injection and 90 min nitrogen purge were executed for each concentration because the hydrogen absorption and release required more time at low concentrations than at high concentrations. The peak heights of signals also showed good reproducibility at these concentrations.

**Figure 4:**
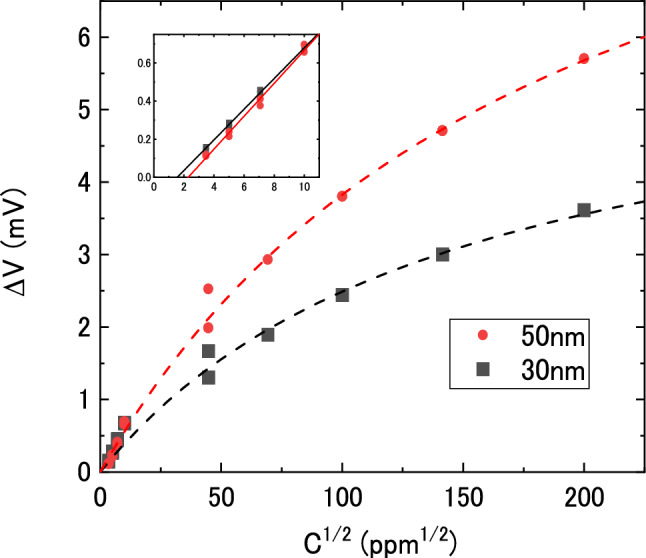
Peak jump heights of MSS output voltage (ΔV) plotted against square root of hydrogen concentration (C^1/2^). Inset: Expansion at low concentrations.

From Figs. 2 and 3, we calculated a signal peak jump height (ΔV) for each injection-purge cycle and plotted ΔV against the square root of hydrogen concentration (C^1/2^) as shown in Fig. 4. The peak jumps are fitted by a Langmuir type (hyperbolic) function. In the inset of Fig. 4, ΔV exhibits a linear relationship to C^1/2^ at low concentrations with crossing about C^1/2^ = 2 (C = 4 ppm). It means that the baselines in Fig. 3 contains slight residual hydrogen, which is difficult to make the residual hydrogen completely 0 in cycles of hydrogen measurements, and the proportional to C^1/2^ means Sieverts’ law. There is no film thickness dependence at low concentrations, due to the difficulty to eliminate residual hydrogen. The theoretical interpretation is given in the next section.

## Theoretical model

This relationship can be explained by either of two different theories. One is the solubility theory (Eq. (1)); the other is the 2sRK model. With the 2sRK model, it is considered that there is dissociative adsorption of hydrogen onto surface site S and absorption into bulk site B. The reaction kinetics can be written as follows:

2 $${\text{S}} + \frac{1}{2}{\text{H}}_{2} { }\begin{array}{*{20}l} {\mathop \rightarrow \limits^{{k_{1 + } }} }\\ {\mathop \leftarrow \limits_{{k_{1 - } }} } \\ \end{array} {\text{ S}}{-}{\text{H }}\begin{array}{*{20}l} {\mathop \rightarrow \limits^{{k_{2 + } }} } \\ {\mathop \leftarrow \limits_{{k_{2 - } }} } \\ \end{array} {\text{ B}}{-}{\text{H}}$$

where S–H corresponds to the status of an adsorbed hydrogen atom on the surface site, B–H corresponds to the status of an absorbed hydrogen atom in the bulk site, and *k*_i_ (*i* = 1 + , 1 − , 2 + , 2 −) corresponds to the reaction-rate constants^15^. The reaction kinetics are expressed as follows:

3 $$\frac{d}{dt}\left[ {{\text{S}}{-}{\text{H}}} \right] = k_{1 + } \left[ {\text{S}} \right]\left[ {{\text{H}}_{2} } \right]^{\frac{1}{2}} - k_{1 - } \left[ {{\text{S}}{-}{\text{H}}} \right] - k_{2 + } \left[ {{\text{S}}{-}{\text{H}}} \right]\left[ {\text{B}} \right] + k_{2 - } \left[ {\text{S}} \right]\left[ {{\text{B}}{-}{\text{H}}} \right]$$

and

4 $$\frac{d}{dt}\left[ {{\text{B}}{-}{\text{H}}} \right] = k_{2 + } \left[ {{\text{S}}{-}{\text{H}}} \right]\left[ {\text{B}} \right] - k_{2 - } \left[ {\text{S}} \right]\left[ {{\text{B}}{-}{\text{H}}} \right]$$

where [S–H] and [S] are the numbers of occupied and empty sites on the surface, and [B–H] and [B] are the numbers of occupied and empty sites in the bulk, respectively. We can define a dimensionless [H_2_] = *P*_H2_/*P*_a_, where *P*_H2_ is the partial pressure of hydrogen and *P*_a_ is atmospheric pressure. From this definition, “[H_2_] = 1” corresponds to a concentration of hydrogen in gas mixture of 100%. Let the maximum number of the adsorbable sites on the surface be *S*_*0*_ and the maximum number of the absorbable sites in the bulk be *B*_*0*_. Then,

5 $$\left[ {\text{S}} \right] + \left[ {{\text{S}}{-}{\text{H}}} \right] = S_{0} .$$

and

6 $$\left[ {\text{B}} \right] + \left[ {{\text{B}}{-}{\text{H}}} \right] = B_{0} .$$

When we assume an equilibrium state in Eqs. (3) and (4), the following equation holds:

7 $$\frac{d}{dt}\left[ {{\text{S}}{-}{\text{H}}} \right] = \frac{d}{dt}\left[ {{\text{B}}{-}{\text{H}}} \right] = 0.$$

From Eqs. (3), (4), and (7),

8 $$\left[ {{\text{S}}{-}{\text{H}}} \right] = \frac{{S_{0} K_{1} \left[ {{\text{H}}_{2} } \right]^{1/2} }}{{1 + K_{1} \left[ {{\text{H}}_{2} } \right]^{1/2} }}$$

and

9 $$\left[ {{\text{B}}{-}{\text{H}}} \right] = \frac{{B_{0} K_{1} K_{2} \left[ {{\text{H}}_{2} } \right]^{1/2} }}{{1 + K_{1} K_{2} \left[ {{\text{H}}_{2} } \right]^{1/2} }}$$

where ${K}_{1}={k}_{1+}/{k}_{1-}$ and ${K}_{2}={k}_{2+}/{k}_{2-}$. The surface adsorption [S–H] obeys the well-known Langmuir’s law. Furthermore, the bulk absorption [B–H] is also expressed as a Langmuir-type function with *K*_1_ and *K*_2_. Thus, we have simply and clearly explained that the hydrogen behaviour into bulk follows a Langmuir-type function, which is same conclusion of the solubility theory (Eq. (1)).

The MSS output signal voltage corresponding to the induced stress of hydrogen absorption, it can be expressed by the following formula:

10 $$V - V_{0} \propto \left[ {{\text{B}}{-}{\text{H}}} \right]$$

where *V*_*0*_ is an initial voltage depending on the residual stress of the sensitive film. The Langmuir-type dependence of hydrogen absorption shown in Fig. 4 is clarified.

We can also discuss Sieverts’ law in terms of this 2sRK model, as has already been proved by Davenport, Dienes and coworkers^16,17^. In the case of [B–H] <  < *B*_*0*_, Eq. (6) can be approximated as:

11 $$\left[ {\text{B}} \right]{ } \approx { }B_{0} .$$

In this case, from Eqs. (3), (4), and (7), the following is obtained instead of Eq. (9),

12 $$\left[ {{\text{B}}{-}{\text{H}}} \right] \approx B_{0} K_{1} K_{2} \left[ {{\text{H}}_{2} } \right]^{1/2} .$$

It can be also obtained directly from Eq. (9) in the case where the concentration of hydrogen is low enough (i.e*.*, [H_2_] <  < 1).

A case that cannot be described by the solubility theory can be described by the 2sRK model. In previous work^7^, we were able to evaluate the initial absorption rate as a function of concentration with a simple Pd film as a sensitive film. The theoretical essence is outlined below. In the case that Reaction 1 is much faster than Reaction 2 (i.e., *k*_1+_, *k*_1−_  >  > *k*_2+_, *k*_2−_), the *k*_2+_ and *k*_2−_ in Eq. (3) can be ignored, thereby being simplified without *k*_2+_, *k*_2−_ as

13 $$\frac{d}{dt}\left[ {{\text{S}}{-}{\text{H}}} \right]{ } \approx { }k_{1 + } \left[ {\text{S}} \right]\left[ {{\text{H}}_{2} } \right]^{\frac{1}{2}} - k_{1 - } \left[ {{\text{S}}{-}{\text{H}}} \right]{ }.$$

Then the differential Eq. (13) can be solved analytically with Eq. (5) and a boundary condition [S–H] = 0 at *t* = 0:

14 $$\left[ {{\text{S}}{-}{\text{H}}} \right]{ } \approx { }\frac{{S_{0} K_{1} \left[ {{\text{H}}_{2} } \right]^{\frac{1}{2}} }}{{1 + K_{1} \left[ {{\text{H}}_{2} } \right]^{\frac{1}{2}} }}\left[ {1 - \exp \{ - \left( {k_{1 + } \left[ {{\text{H}}_{2} } \right]^{\frac{1}{2}} + k_{1 - } } \right)t\} } \right].$$

Since *k*_1+_ and *k*_1−_ are large enough and the exponential function in Eq. (14) rapidly approaches 0, [S‑H] can be regarded as Langmuir adsorption. If we consider the initial process of absorption into the bulk (i.e., [B‑H]$\approx$ 0), then Eq. (4) becomes,

15 $$\frac{d}{dt}\left[ {{\text{B}}{-}{\text{H}}} \right]{ } \approx { }k_{2 + } \left[ {{\text{S}}{-}{\text{H}}} \right]\left[ {\text{B}} \right] = \frac{{S_{0} B_{0} K_{1} k_{2 + } \left[ {{\text{H}}_{2} } \right]^{\frac{1}{2}} }}{{1 + K_{1} \left[ {{\text{H}}_{2} } \right]^{\frac{1}{2}} }}.$$

This equation indicates that when Langmuir adsorption takes place quickly on the surface, the absorption rate at the initial process is proportional to the hydrogen density on the surface. As a result, a Langmuir-type function appears in the absorption rate.

## Summary and conclusion

We showed experimentally that the process of hydrogen absorption into an amorphous Pd alloy film reaches saturation very quickly with little hysteresis. It was explained by each of the solubility theory and the 2sRK theory that the amount of absorbed hydrogen is a Langmuir-type (hyperbolic) function of the square root of the hydrogen partial pressure (or hydrogen concentration) instead of Sieverts’ law. The reason why the two theories draw the same conclusion is that they do not suppose the common assumption that the number of absorbable sites in the bulk is infinite compared to the number of absorbed hydrogens (i.e., Eq. (6) holds). This means that Sieverts’ law may not be suitable when evaluating the absorption of fine particles or very thin films. The 2sRK model may be useful in studying the phenomena of hydrogen adsorption and absorption for nanomaterials or nanotechnology. For example, in experiments with high-concentration hydrogen permeation, the deviation from Sieverts’ law often becomes an important aspect. An approximate term ${{p}_{H2}}^{n} (n\ne \frac{1}{2})$ is often used in place of the square root of hydrogen partial pressure (${{p}_{H2}}^{1/2}$), but there is no rationale^18^. Some of these issues may be solved by the assumption of a Langmuir-type dependency.

## References

[1] Broom DP (2011). Hydrogen Storage Materials.

[2] Ockwig NW, Nenoff TM (2007). Membranes for hydrogen separation. Chem. Rev..

[3] Hübert T, Boon-Brett L, Black G, Banach U (2011). Hydrogen sensors—A review. Sensors Actuators, B Chem..

[4] Okuyama S, Mitobe Y, Okuyama K, Matsushita K (2000). Hydrogen gas sensing using a Pd-coated cantilever. Jpn. J. Appl. Phys..

[5] Fowler RH, Smithells CJ (1937). A theoretical formula for the solubility of hydrogen in metals. Proc. R. Soc. Lond. Ser. A Math. Phys. Sci..

[6] Lacher JR (1937). A theoretical formula for the solubility of hydrogen in palladium. Proc. R. Soc. Lond. Ser. A Math. Phys. Sci..

[7] Yakabe T, Imamura G, Yoshikawa G, Kitajima M, Itakura AN (2020). Hydrogen detection using membrane-type surface stress sensor. J. Phys. Commun..

[8] Yoshikawa G, Akiyama T, Gautsch S, Vettiger P, Rohrer H (2011). Nanomechanical membrane-type surface stress sensor. Nano Lett..

[9] Sakamoto Y, Takashima I (1996). Hysteresis behaviour of electrical resistance of the Pd-H system measured by a gas-phase method. J. Phys. Condens. Matter.

[10] Darmadi I, Nugroho FAA, Langhammer C (2020). High-performance nanostructured palladium-based hydrogen sensors—current limitations and strategies for their mitigation. ACS Sensors.

[11] Kajita S, Yamaura SI, Kimura H, Inoue A (2010). Hydrogen sensing ability of Pd-based amorphous alloys. Sensors Actuators, B Chem..

[12] Hayashi Y, Yamazaki H, Ono D, Masunishi K, Ikehashi T (2018). Investigation of PdCuSi metallic glass film for hysteresis-free and fast response capacitive MEMS hydrogen sensors. Int. J. Hydrogen Energy.

[13] Hayashi Y (2020). Effects of poisoning gases on and restoration of PdCuSi metallic glass in a capacitive MEMS hydrogen sensor. Int. J. Hydrogen Energy.

[14] Sakurai, J., Hata, S. & Shimokohbe, A. Reduction of Electrical Resistivity in PdCuSi Thin Film Metallic Glass. *Abstr. ATEM Int. Conf. Adv. Technol. Exp. Mech. Asian Conf. Exp. Mech.* **2**, (2003).

[15] Conrad H, Ertl G, Latta EE (1974). Adsorption of hydrogen on palladium single crystal surfaces. Surf. Sci..

[16] Pick MA, Davenport JW, Strongin M, Dienes GJ (1979). Enhancement of hydrogen uptake rates for Nb and Ta by thin surface overlayers. Phys. Rev. Lett..

[17] Davenport JW, Dienes GJ, Johnson RA (1982). Surface effects on the kinetics of hydrogen absorption by metals. Phys. Rev. B.

[18] Suzuki A, Yukawa H (2020). A review for consistent analysis of hydrogen permeability through dense metallic membranes. Membranes (Basel)..

